# *In Vivo* Mouse Models for Hepatitis B Virus Infection and Their Application

**DOI:** 10.3389/fimmu.2021.766534

**Published:** 2021-10-29

**Authors:** Yanqin Du, Ruth Broering, Xiaoran Li, Xiaoyong Zhang, Jia Liu, Dongliang Yang, Mengji Lu

**Affiliations:** ^1^ Institute for Virology, University Hospital Essen, University of Duisburg-Essen, Essen, Germany; ^2^ Department of Gastroenterology and Hepatology, University Hospital Essen, University of Duisburg-Essen, Essen, Germany; ^3^ State Key Laboratory of Organ Failure Research, Guangdong Provincial Key Laboratory of Viral Hepatitis Research, Department of Infectious Diseases, Nanfang Hospital, Southern Medical University, Guangzhou, China; ^4^ Department of Infectious Diseases, Union Hospital, Tongji Medical College, Huazhong University of Science and Technology, Wuhan, China

**Keywords:** hydrodynamic injection, viral vector, transgenic mouse, liver humanized mouse, hepatitis B virus

## Abstract

Despite the availability of effective vaccination, hepatitis B virus (HBV) infection continues to be a major challenge worldwide. Research efforts are ongoing to find an effective cure for the estimated 250 million people chronically infected by HBV in recent years. The exceptionally limited host spectrum of HBV has limited the research progress. Thus, different HBV mouse models have been developed and used for studies on infection, immune responses, pathogenesis, and antiviral therapies. However, these mouse models have great limitations as no spread of HBV infection occurs in the mouse liver and no or only very mild hepatitis is present. Thus, the suitability of these mouse models for a given issue and the interpretation of the results need to be critically assessed. This review summarizes the currently available mouse models for HBV research, including hydrodynamic injection, viral vector-mediated transfection, recombinant covalently closed circular DNA (rc-cccDNA), transgenic, and liver humanized mouse models. We systematically discuss the characteristics of each model, with the main focus on hydrodynamic injection mouse model. The usefulness and limitations of each mouse model are discussed based on the published studies. This review summarizes the facts for considerations of the use and suitability of mouse model in future HBV studies.

## Introduction

Hepatitis B virus (HBV) is a prototypical member of the *Hepadnaviridae* family (1). Despite the availability of an effective vaccine, HBV infection remains a global health issue that affects approximately 3.5% of the global population, with around 257 million chronically infected people worldwide (2). These patients have a high risk of developing into end-stage liver diseases such as cirrhosis and hepatocellular carcinoma (HCC). Current treatment options of chronic HBV infection include antiviral cytokine pegylated interferon (IFN) alpha, and the HBV polymerase inhibitors nucleos(t)ide analogues (NAs). However, the available treatment regimens rarely clear HBV in chronically infected patients.

The host spectrum of HBV is very narrow; only few primates such as chimpanzees (3) and Mauritian cynomolgus monkeys (4) are susceptible to HBV infection. Tree shrews (*Tupaia belangeri*) have also been reported to be susceptible to HBV infection, but it is transient and mild (5). Other members of the *Hepadnaviridae* family, such as duck hepatitis virus and woodchuck hepatitis virus have been used for studies on HBV replication and pathogenesis in their own hosts (6–9). However, such specific virus-host systems are significantly different from the situation in humans with HBV infection (10). Moreover, the lack of essential reagents for studies in these hosts, ethical restrictions, and high costs for animal maintenance and care strongly limit the use of these animal models. Therefore, easy-to-handle HBV models based on laboratory mouse strains are important animal models for HBV research.

HBV mouse models have evolved and greatly improved research progress. In this review, we summarize the currently available mouse models for HBV research, including hydrodynamic injection (HDI), viral vector-mediated transfection, HBV recombinant covalently closed circular DNA (rc-cccDNA), transgenic, and liver humanized mouse models. We systematically discuss the characteristics, usefulness, and limitation for each model.

## HDI Mouse Model

HDI is an efficient procedure for delivering genetic materials to the mouse liver. During this procedure, a large volume of liquid containing HBV DNA plasmid is injected through the mouse tail vein by pressure within few seconds. The high pressure permeabilizes the capillary endothelium and generates “pores” in the plasma membrane of surrounding hepatocytes, through which DNA may reach the intracellular space (11). Then the retained HBV plasmid DNA initiates HBV replication by transcription of pregenomic RNA and other HBV mRNAs, followed by the formation of HBV replication intermediates and expression of HBV proteins. The standard procedure of HDI requires injection of liquid equal to 8–10% mouse weight (g) within 5–8 s (12, 13). Different vectors containing replication-competent HBV genomes could be used for this purpose, including an adeno-associated virus/HBV 1.2 plasmid (pAAV/HBV1.2) with a 1.2-fold overlength HBV genotype A genome (13), pcDNA 3.1(+)-HBV1.3C with a 1.3-fold HBV genotype C genome (14), and pSM2 plasmid with a head-to-tail tandem dimeric HBV genome (15).

### Factors Influencing HBV Persistence in the HDI Mouse Model

The HDI HBV mouse model can be used to establish transient or persistent HBV replication with HBsAg secretion into the peripheral circulation from 1 week to more than 6 months. HBV persistence in this model is determined by different factors, including the genetic background of mouse strains, age, gender, plasmid backbone, and the dosage of plasmid (**Table 1**). The genetic background plays a key role in HBV exposure outcome. It has been reported that 40–60% C3H/HeH and C57BL/6 mice remain HBV-positive up to 6 months after HDI of 10 μg pAAV/HBV1.2 (13, 18–20). In contrast, BALB/cJ, FVB/NJ, NOD/ShiLtJ, and 129×1/SvJ mice that received the same plasmid injection rapidly clear HBV within 3-5 weeks post injection (wpi). Even mice with the same background but different substrains display different outcomes after HDI of HBV plasmid. In C57BL/6N mice, HBsAg and HBV DNA rapidly decline below the limit of detection within 8 weeks, while HBsAg remains detectable in C57BL/6J mice at 26 wpi (21).

**Table 1:** Factors influence HBV persistence in HDI mouse model.

Authors	Mouse background/gender/age	Plasmid backbone, dosage	Persistent of HBV antigens or replicative intermediates
**Yang et al. (** 12 **)**	B10.D2 and CB17 NOD/Scid (6-9 weeks)	13.5 μg pT-MCS-HBV1.3 and 4.5 μg pCMV-SB	HBsAg, HBeAg disappeared at 7 dpi; HBcAg 1 dpi (6%), 7 dpi (4%)
**Huang et al. (** 13 **)**	C57BL/6 (male, 6-8 weeks)	10 μg, pAAV/HBV1.2	80% mice HBsAg positive at week 5; 40% HBsAg positive > 6 month; replicative intermediates remained detectable at 22 dpi
	BALB/c (male, 6-8 weeks)	10 μg, pAAV/HBV1.2	HBsAg disappeared from week 2; replicative intermediates decreased from 14 dpi
	C57BL/6 (male, 6-8 weeks)	10 μg, pGEM4Z/HBV1.2,	HBsAg disappeared within 3 weeks
**Li et al. (** 14 **)**	C57BL/6 (male, 6-8 weeks)	15 μg pAAV/HBV1.3, pcDNA3.1(+)-HBV1.3	HBsAg disappeared within 8 weeks after injection of 15 μg pAAV/HBV1.3; 60% mice were HBsAg-positive at week 20 after injection of 15 μg pcDNA3.1(+)-HBV1.3
**Li et al. (** 16 **)**	C57BL/6 (male, 6-8 weeks)	5, 10, or 100 μg pAAV/HBV1.2	80% mice were HBsAg positive for more than 6 months (5 μg); 40% mice were HBsAg positive for more than 6 months (10 μg); HBsAg was cleared at week 5 (100 μg);
	BALB/c (male, 6-8 weeks)	1 or 5 μg, pAAV/HBV1.2	20% HBsAg positive > 3 month (1μg); 60% HBsAg positive > 3 month (5 μg)
**Wang et al. (** 17 **)**	C57BL/6 (male, 5-6 weeks)	6 or 20 μg, pAAV/HBV1.2	100% HBsAg positive at 6 wpi (6 μg); HBsAg disappeared at 4 wpi (20 μg);
		6 μg pAAV/HBV1.2 + 14 μg pAAV/control	HBsAg disappeared at 4 wpi
**Chou et al. (** 18 **)**	BALB/cJ, FVB/NJ, NOD/ShiLtJ, 129×1/SvJ (male, 6 weeks)	10 μg pAAV/HBV1.2	HBsAg rapidly disappeared within 4 weeks after injection
	C3H/HeN, C57BL/6, DBA/2J, CBA/caJ (male, 6 weeks)	10 μg pAAV/HBV1.2	40% C57BL/6, 90% C3H/HeN, 75% DBA/2J, 100% CBA/caJ remained HBsAg-poitive at 8 wpi
	C3H/HeN, C57BL/6, DBA/2J, CBA/caJ (male, 12 weeks)	10 μg pAAV/HBV1.2	HBsAg disappeared at 5 wpi in C3H and DBA/2J mice; 12-week-old C57BL/6 mice accelerated HBsAg clearance compared to younger mice
**Peng et al. (** 19 **)**	C3H/HeN (male, 5-6 weeks)	10 μg pAAV/HBV1.2	HBsAg persisted for up to 46 weeks
**Yuan et al. (** 20 **)**	AAVS1 (female and male, 6-8 weeks)	10 μg pAAV/HBV1.2	Male mice express higher levels of HBsAg, HBeAg than female mice
**Kosinska et al. (** 15 **)**	C57BL/6 (female and male, 9-10 weeks)	10 μg pSM2	Male mice express higher levels of HBsAg, HBeAg than female mice

Dpi, days post injection; wpi, weeks post injection.

HBV persistence differs in mice of different ages or gender after HDI. 12-week-old adult C3H/HeN mice have been shown to clear HBV within 6 wpi; however, 50% of their younger counterparts (6-week-old) remain HBsAg-positive at 26 wpi (18). The same study suggested that adult mice possess mature gut microbiota that stimulates liver immunity, resulting in rapid HBV clearance, while younger mice are immune-tolerant through the Toll-like receptor (TLR)-4 dependent pathway (18). Another study using AAVS1 mice demonstrated that male AAVS1 mice receiving 10 μg pAAV/HBV1.2 express higher HBsAg and HBeAg levels than female mice (20). Similarly, male C57BL/6 mice that received 10 μg pSM2 plasmid expressed higher levels of HBV proteins than their female counterparts (15). Mechanistically, it is due to high frequency of regulatory T cells and low response of HBV-specific T cells in male mice (15).

The plasmid backbone also affects HBV persistence in mice after HDI. In contrast to pAAV/HBV1.2, injection of pGEM4Z with the same 1.2-fold HBV genotype A genome into C57BL/6 mice produces only transient antigenemia, and HBsAg rapidly disappears within 3 weeks (13). In line with this, mice injected with 15 μg pAAV/HBV1.3 containing 1.3-fold HBV genotype C genome clear the HBsAg within 8 weeks while 60% mice remain HBsAg-positive at week 20 post HDI of pcDNA3.1(+)-HBV1.3 (14).

Finally, the doses of plasmid backbone or HBV genome also greatly impact HBV persistence in HDI model. Approximately 40-80% C57BL/6 mice that receive 5 or 6 μg pAAV/HBV1.2 develop HBsAg persistence up to 6 months, while a high dose of 100 μg pAAV/HBV1.2 results in HBsAg clearance within 4-5 weeks (16, 17). Similarly, 20 μg pAAV/HBV1.2 or 6 μg pAAV/HBV1.2 plus 14 μg pAAV/control plasmid also results in HBV clearance within 4 weeks (17).

These data highlight the need for researchers to carefully choose mouse strains and plasmid doses when using this model in HBV research.

### Application of the HDI Mouse Model for Studies on Immune Responses During Virus Clearance

There is a consensus that adaptive immune responses, especially those of virus-specific T cells, play an essential role in mediating intrahepatic HBV clearance (22). In acute HBV infection in humans, viral clearance is associated with the development of a vigorous multispecific CD8+ T cell response and an acute necroinflammatory liver disease; it was therefore assumed that HBV clearance is principally mediated by virus-specific major histocompatibility complex class I-restricted cytotoxic T lymphocytes (CTLs) (23). The immune effectors required for HBV clearance were screened in an HBV HDI mouse model, which also demonstrated that CD8+ T cells are the key cellular effectors mediating hepatic HBV clearance (24). Consistent with these findings, chronic HBV infection is characterized by weak or undetectable HBV-specific CD8+ T cell responses and the presence of functionally exhausted HBV-specific CD8+ T cells that are unable to clear the virus (25–27).

The use of mouse models allows detailed analysis of immune responses to HBV *in vivo*. Several studies employed HDI mouse models to investigate immune responses to HBV and their relationship with HBV clearance. An acute HBV replication mouse model was established by HDI of pT-MCS-HBV1.3 and pCMV-SB. A variety of immunodeficient mouse strains have been used to determine the roles of different types of immune cells in HBV clearance in this model; the results showed that natural killer (NK) cell, type I IFN, CD4, and CD8, but not B cells, contribute to HBV clearance (24). Similarly, Tseng et al. showed that the molecules involved in innate immunity, including IFN-α/β receptor (IFNAR), RIG-I, MDA5, Myd88, NLRP3, ASC, and interleukin-1 receptor (IL-1R), are not essential for HBV clearance in the mouse model (28). Another study using a panel of mouse strains lacking specific innate immunity components demonstrated that HBV-specific T cell responses are functionally impaired in TLR signaling-deficient mice, thus allowing enhanced and prolonged HBV replication and expression (29). In line with this, when blocking the type I IFN receptor or TLR7 signaling pathway, CD8^+^ T cell activation and HBV clearance are significantly impaired (30).

The immune factors that suppress or contribute to HBV persistence could be also found by comparing HBV HDI mouse models that mimic acute-resolving and chronic HBV infection. By comparing C57BL/6N and C57BL/6J mice, Wang et al. found that regulatory T (Treg) cells suppress the follicular helper T (Tfh) cells response to HBsAg in C57BL/6J mice and thus lead to persistent HBV infection (21). Importantly, this is consistent with the impaired Tfh-cell response to HBsAg in patients with chronic HBV infection (21). Consistently, depletion of Tregs or inhibition of their function with a blocking antibody against cytotoxic T-lymphocyte-associated protein 4 (CTLA4) restored the Tfh-cell response to HBsAg both in the HBV-persistent mice and patients with chronic HBV infection (21). In line with this, our group demonstrated that intrahepatic HBV replication resulted in increased intrahepatic Treg infiltration and thus limited intrahepatic anti-HBV CD8+ T cell responses (31). We further demonstrated that the suppression of anti-HBV CTL function by Tregs is gender related, which nicely explains the gender-related differences in HBV infection outcomes in humans (15). It has also been reported that the chronic HBV replication mouse model established by HDI of pAAV/HBV1.2 is unable to respond to HBsAg vaccination, which is attributed to IL-10 secretion by Kupffer cells (KCs) (32). Moreover, extracellular vesicles secreted by HBV-infected cells from patients with HBV exert immunosuppressive functions and lead to HBV persistence in the HDI model (33). Recently, we demonstrated that during acute-resolving HBV infection in HDI model using pSM2 plasmid, the liver produces increased amounts of matrix metalloprotease (MMP)2, which, together with MMP9, mediate membrane CD100 shedding from the surface of T cells and NK cells in the secondary lymphoid organs and increase serum soluble CD100 levels (34). By interacting with CD72, sCD100 induces the activation of antigen-presenting cells in the spleen and liver in the HDI mice, thus promoting the intrahepatic anti-HBV CD8 T cell response and virus clearance (34).

### Other Applications of the HDI Mouse Model

Immune therapeutic strategies that aim to restore the antiviral function of HBV-specific CD8^+^ T cells have been intensively examined in HBV HDI mouse models that mimic chronic HBV infection. To mimic patients with chronic hepatitis B, the persistent HBV replication mouse models are usually established by HDI of 6-10 μg pAAV/HBV1.2 plasmid into 5-to-6-week-old male C57BL/6 or C3H mice (35, 36). Our group previously demonstrated that intravenous administration of TLR3 ligand Polyinosinic:polycytidylic acid [poly(I:C)] led to HBV clearance, which was associated with increased CD8^+^ T cell infiltration in the liver in an IFN-γ- and CXCR-3-dependent manner (37). Furthermore, we also showed that the NOD1 ligand, D-glutamyl-meso-diaminopimelic acid, enhanced the antigen-presenting function of liver sinusoidal endothelial cells to promote HBV-specific CD8^+^ T cells and HBV clearance in an HBV-persistent HDI mouse model (35). Therapeutic strategies targeting immune checkpoint programmed death-1/programmed death ligand-1 to restore the antiviral function of HBV-specific CD8^+^ T cells were also examined in an HBV persistent HDI mouse model (38).

HDI mouse models are also widely used to test antiviral agents such as lamivudine (14), RNA interference targeting HBV replicative intermediates (39), and the HBV genome-specific guide RNA-mediated clustered regularly interspaced short palindromic repeats (CRISPR)/Cas9 system (40). Anti-HBV vaccines such as cytomegalovirus-based vaccines (41) were also tested in this model.

Another advantage of the model is that plasmids of different HBV genotypes and variants or mutants can be constructed and injected into the mice. Therefore, they can be used for testing antiviral effects against mutated HBV (42) and exploring the influence of HBV genome strain on HBV persistence, as well as its underlying mechanisms (14, 43).

### Limitations of the HDI Mouse Model

Although HDI mouse models are widely used to investigate immune responses and evaluate antiviral compounds, the limitations should be carefully considered. Firstly, the HDI method is stressful for animals as it involves injecting a large volume of liquid, and it also causes significant liver damage in the first few days after injection (10). Secondly, the non-HBV immune response elicited by the plasmid backbone should also be considered. Thirdly, mouse hepatocytes are extremely inefficient in forming cccDNA from rcDNA, resulting in a general absence of cccDNA (44). Fourthly, intrahepatic transfection efficiency is only ~6% in this model (12), much lower than in HBV-infected patients in the real world. Finally, due to the lack of the entry receptor in mice, this is not a natural infection model, so it cannot be used to study the entire infection process. Considering the huge differences between HDI mouse model and patients with HBV infection, researchers should be careful when evaluating the findings about the therapeutic value of immunological and pharmacological interventions against acute and chronic HBV infection in hydrodynamic transfection systems.

There are other aspects to consider when interpreting results from HDI mouse models. When modeling acute HBV infection, the levels of HBsAg and HBV DNA decline quickly in the initial phase. This also occurs under treatment with various of immunosuppressive agents (45), suggesting that this process is not immune-related and that the results obtained in such a situation should be interpreted with care. Although HBV can persist in the host and maintain its replication over a prolonged period, the immunological mechanisms leading to persistence are primarily related to lack of recruitment and intrahepatic accumulation of activated T cells to HBV. Intrahepatic immune activation by TLR ligands result in immune cell infiltration and HBV clearance (37). A major issue is the lack of inflammation in mice with persistent HBV replication, resulting in insufficient recruitment of immune cells into the liver. Thus, HDI mouse models may be useful for investigating host immune responses and viral clearance or persistence; however, a direct comparison with acute and chronic HBV infection in humans is not always possible.

## Viral Vector-Mediated Transfection Mouse Model

Viral vector-mediated transfection mouse model is based on viral transduction by intravenous injection hepatotropic viral vectors containing 1.2- to 1.3-fold HBV genomes, including Adenovirus (Ad) (46–48), and Adeno-associated virus (AAV) (49–51). In this model, HBV genome is inserted to the genome of viral vectors, which initiates HBV replication and secretion of infectious HBV virions. Both vector models express HBV replication intermediates, and the dose of Ad-HBV directly correlates with the outcome of HBV persistence (52). AAV belongs to the family *Parvoviridae*, which is characterized by site-specific integration, natural deficiency, and low immunogenicity. AAV has a variety of serotypes, each of which has organ specificity, making it an ideal gene-targeting vector. Studies have shown that the liver transduction efficiency of AAV8 is stronger than that of AAV1, 5, and 6 serotypes, indicating that AAV8 is highly hepatotropic (53). This feature is favorable for the application of AAV8 in HBV-related studies.

### Applications of the Viral Vector-Mediated Transfection Mouse Model

Both Ad-HBV and AAV-HBV transfection mouse models can establish persistent HBV replication for more than 3 months or even more than 1 year (46, 52, 54). Notably, only low doses of Ad-HBV have been reported to establish such persistent HBV replication (46). The high doses of Ad-HBV can induce acute-resolving hepatitis, which is partially due to strong non-HBV immune responses induced by the vector itself (55, 56). Nevertheless, this permits the investigation of mechanisms of immune-mediated viral clearance, including the role of NK cells (57) and intrahepatic CTLs (58).

Compared with Ad-HBV, AAV-HBV vectors have minimal AAV genomes with only the essential AAV inverted terminal repeat sequence that is responsible for viral packaging and does not encode any AAV viral proteins. Thus, this vector has a clean background and does not induce an obvious non-HBV immune response (59), making it a more suitable tool to establish persistent HBV infection (10). More importantly, the AAV-HBV mouse model allows more efficient and homogeneous HBV transduction than the HDI model with ~60% of hepatocytes expressing HBcAg 12 weeks after injection (49).Serum HBsAg in AAV-HBV mice remains at relative high levels even in 3 or 4 month (**Table 2**). Therefore, the AAV-HBV mouse model is preferred to evaluate therapeutic vaccines for chronic infection (61) and the antiviral effects of TLR agonists like the TLR9 ligand CpG (50). It is worth mentioning that Prof. Xiaobing Wu’s group in Shanghai developed a persistent HBV replication mouse model by transduction of recombinant AAV8 (rAAV8)-HBV 1.3 into C57BL/6 mice (62). This model is also used to evaluate antiviral effects of NAs such as entecavir and lamivudine (63). Moreover, two studies reported fibrosis in HBV mice transfected with AAV8-HBV1.2, which is one of the few replicative models that can be used to study the mechanism of liver fibrosis in the course of chronic hepatitis B (64, 65). Lucifora et al. reported that cccDNA can be detected by Southern blot in an AAV-HBV model (66). However, it remains uncertain whether AAV-HBV generates cccDNA in murine hepatocytes through rcDNA, the natural precursor of cccDNA.

**Table 2:** Serum HBsAg levels among different chronic HBV mouse model.

Authors	Mouse background	Mouse model	HBsAg positive rate	HBsAg levels
**Huang et al. (** 13 **)**	C57BL/6 (male, 6-8 weeks)	HDI model 10 μg pAAV/HBV1.2	80% at 5 wpi	10000 ng/ml at 1 wpi; 100-1000ng/ml at 5 wpi
**Li et al. (** 14 **)**	C57BL/6 (male, 6-8 weeks)	HDI model 15 μg pcDNA3.1(+)-HBV1.3	60% at 20 wpi	OD450: 3.0-3.5 at 1 wpi; ~1.5 at 20 wpi
**Li et al. (** 16 **)**	C57BL/6 (male, 6-8 weeks)	HDI model 5μg pAAV/HBV1.2	80% at 24 wpi	OD450: 2.5-3.0 at 1 wpi 1.0-2.5 at 24 wpi
**Wang et al. (** 17 **)**	C57BL/6 (male, 5-6 weeks)	HDI model 6 μg pAAV/HBV1.2,	100% at 6 wpi	1000 ng/ml at 1 wpi; 100-1000 ng/ml at 6 wpi
**Peng et al. (** 19 **)**	C3H/HeN (male, 5-6 weeks)	HDI model 10 μg pAAV/HBV1.2	90% at 46 wpi	2000-3000 IU/ml at beginning; 60-100 IU/ml at 46 wpi
**Huang et al. (** 46 **)**	n.a	Ad-HBV vector Infected with 10^8^ infectious unites of Ad-HBV	n.a	10000-100000 S/CO at 1 wpi; ~ 100 S/CO at day 100 after infection
**Dion et al. (** 49 **)**	HLA-A2/DR1 mice (male, 6-8 weeks)	AAV2/8-HBV vector Infected with 5×10^10^vg	n.a	~ 100 μg/ml at 2 wpi ~ 80 μg/ml at 16 wpi
**Yang et al. (** 50 **)**	C57BL/6 (male, 6-8 weeks)	AAV-HBV vector Infected with 1×10^11^vg	n.a	2000-3000 ng/ml at 2 wpi and 12 wpi
**Yan et al. (** 60 **)**	C3H (male, 4-6 weeks)	Rc-ccc DNA model HDI of 10 μg of HBVcircle	100% at 7 wpi	10000 ng/ml at 1 wpi; 1000-10000 ng/ml at 7 wpi

HDI, hydrodynamic injection; HLA-A2/DR1, HLA-A*0201/DRB1*0101-transgenic, H-2 class I/class II knockout (KO) mice; n.a, not available; S/CO, signal to control ratio; wpi, weeks post injection.

### Limitations of the Viral Vector-Mediated Transfection Mouse Model

As adenoviral vectors have large genomes and encode numerous non-HBV viral products, it is technically challenging to interpret the HBV-related immune response and pathogenesis in the Ad-HBV transfection model (55, 56). Moreover, except for the observed liver fibrosis in AAV8-HBV1.2-transfected mice mentioned above, Ad-HBV and AAV-HBV mice do not usually develop pathological changes in the liver. Although rAAV8-HBV1.3 can to some extent achieve persistent HBV replication after transfection, it cannot stimulate HBV-specific humoral immunity (67). This may be due to immune tolerance induced by the AAV8 vector (67). Moreover, similar to the HDI approach, this model does not represent real infection. For these reasons, it is controversial whether this model can be used to study HBV immunology, which limits its application.

## HBV RC-cccDNA Mouse Model

The stable cccDNA pool is a barrier to HBV eradication in patients. However, one common disadvantage of the HDI injection and virus-vector transduction mouse models is that HBV cccDNA is rarely and not convincingly detected, suggesting that the intracellular recycling pathway is severely impaired in mouse hepatocytes (68). To address cccDNA related issues, a recombinant cccDNA (rc-cccDNA) mouse model was developed. In 2014, Qiang Deng’s group first reported that coinjection of a plasmid containing double-LoxP flanked HBV DNA sequence and a plasmid expressing Cre recombinase induced rc-cccDNA generation in the murine liver (69). This rc-cccDNA is similar to the real HBV cccDNA that acts as the template producing mature HBV virus. However, the plasmid backbone after excision remained in the mouse liver and evoked a non-HBV immune response, which critically influenced rc-cccDNA stability and HBV antigenemia *in vivo* (69). To overcome this issue, the same group developed an improved version of the cccDNA mouse using a replication-defective recombinant adenoviral vector to deliver linear HBV genome into Cre transgenic mice (70). HBV persists more than 62 weeks in this model, and sustained necroinflammatory response and fibrosis can be observed in the liver, which is analogous to the progressive pathology of clinical hepatitis (70).

Alternatively, a hydrodynamic-injected recombinant HBV minicircle cccDNA generated from a genetically engineered *Escherichia coli* strain also formed authentic cccDNA-like molecules in murine hepatocytes and achieved high-level, persistent HBV replication in C3H mice (60). Another group recently developed rc-cccDNA to mimic cccDNA using HDI of a Cre/LoxP-HBV plasmid into NOD-scid IL1Rg^null^ mice (71). In their system, HBx-mutant and green fluorescent protein reporter plasmids are created by these cis-Cre/LoxP-HBV plasmids to further probe cccDNA biology and develop antiviral strategies against cccDNA (71).

In the rc-cccDNA mice, HBV replication and observed phenotypes are entirely driven by cccDNA-like viral genome, making it a more biologically relevant model for testing antiviral agents targeting cccDNA, such as CRISPR/Cas9 nuclease, as well as small molecules specifically silencing or destabilizing cccDNA (72, 73). Nevertheless, the rc-cccDNA does not have the fully identical characteristics of cccDNA formed from rcDNA. These rc-cccDNA systems also do not overcome the limitation of impaired intracellular recycling pathway in mouse hepatocytes. Therefore, the application of this system is still limited.

## Transgenic Mouse Models

In the 1980s, HBV transgenic mouse models were generated with embryo microinjection technology. These transgenic lineages express one of the HBV gene products under the control of the native viral regulatory elements or cell-specific promoters (74). The insertion of overlength HBV genomes supports viral replication, including particle production and infectious virion release (3, 75, 76). Transgenic models have substantially contributed to the understanding of molecular virus-host interactions and the biology of HBV-related immunology and pathogenesis. They can be separated into single HBV-protein transgenic mice and full-genome transgenic mice.

### Single-Protein Transgenic Mice

The HBV protein-transgenic mice express HBV proteins such as HBsAg (77, 78), HBcAg (79), HBeAg (80), or HBx (81). These models can be used to study the virology and oncogenic potential of these HBV proteins.

In 1985, Chisari et al. developed the first HBV transgenic mice that express HBsAg (77). It was initially suggested that this model did not exhibit signs of pathology. Later, the same group described that increased expression of large HBsAg, rather than the small and middle HBsAg proteins, inhibits the secretion of HBsAg-containing particles (78). Furthermore, HBsAg accumulation in the endoplasmic reticulum of hepatocytes leads to continuous inflammation and liver injury, followed by HCC development (82). The same HBs-transgenic mice were crossed to C57BL/6 and BALB/c, which revealed that HBsAg protein-induced liver injury depends on host genetic background (83). It was recently shown that methylation of specific CpG sites controls gene expression in these HBs-transgenic mice, resulting in decreased cell stress and improved liver integrity (84). Although pathological aspects of HBsAg could be revealed with this model, the intracellular accumulation does not reflect the natural function of HBsAg in HBV-infected humans.

To address HBV biology and immunological aspects, HBeAg-transgenic mice were generated to investigate the role of immunological tolerance in chronic infection of newborns (80). Both HBeAg and HBcAg are expressed in this model. It has been suggested that HBeAg secretion may be one of the viral strategies for HBV persistence after perinatal infection (80). Another HBcAg-transgenic mouse model was used to examine factors that influence the intracellular localization of nucleocapsid particles in hepatocytes; it demonstrated that nucleocapsids can form *de novo* within the nucleus, and the reformed nucleocapsid cannot be transported across the intact nuclear membrane (79). Knowledge about the functional and structural significance of HBcAg and HBeAg could be gained with these models.

Mice transgenic for HBx protein have yielded controversial results regarding its oncogenic potential. Early reports showed that HBx-expressing mice develop HCC (81, 85, 86). However, others reported contradicting findings that HBx alone is not sufficient for inducing HCC development (87–89). Hepatocarcinogenesis has been directly compared in HBsAg and HBx knock-in transgenic mice. HBsAg and HBx genes were integrated into the mouse p21 locus using homologous recombination. Although p21-HBx transgenic mice developed HCC at the age of 18 months, p21-HBsAg transgenic mice have developed HCCs 3 months earlier. Herein, the expression of genes related to metabolism and genomic instability largely resembled the molecular changes during HCC development in humans (90). However, hepatocarcinogenesis in HBV-transgenic mice occurs in a cirrhosis-free condition and does not reflect the common situation in HCC-developing patients with chronic hepatitis B infection.

### Full-Genome Transgenic Mice

Single-HBV protein transgenic mice facilitated investigation of the assembly, secretion, immune responses, and functionality of these proteins *in vivo*. However, HBV-full genome transgenic mice support the whole HBV life cycle *in vivo*. The integrated HBV genome includes terminally redundant DNA representing a 1.3 overlength HBV genome leading to efficient viral replication, including rcDNA synthesis from pregenomic RNA, viral assembly, and production of infectious HBV particles that are morphologically indistinguishable from human-derived virions (76). Due to self-tolerance, full-genome transgenic mice do not suffer from T cell-related liver injury. The lack of hepatitis indicates that HBV viral proteins or HBV itself is not cytopathic in transgenic mice. Adoptive transfer of HBV-specific cytotoxic T cells caused acute liver disease and thus resulted in temporal clearance of HBsAg (91, 92). These HBV replication-competent transgenic mice have been extensively used to evaluate antiviral drugs that hinder HBV replication, including small interfering RNAs (93, 94); cytokines (95, 96); and various polymerase inhibitors such as adefovir dipivoxil (97), lamivudine (98), and entecavir (99). Thus, cellular, molecular pathogenic, and antiviral mechanisms occurring in acute viral hepatitis have been recapitulated in full-genome transgenic mice.

### Limitation of Transgenic HBV Models

All lineages of HBV transgenic mice generated to date are immunologically tolerant to viral antigens, particularly at the T-cell level, and they do not develop acute or chronic hepatitis. Therefore, these models do not develop HBV-related liver diseases including liver injury and progressive fibrosis and cirrhosis. Due to the transgenic origin of HBV replication, these mice do not have the potential of viral clearance (100). Another restriction is the lack of cccDNA in these full-genome transgenic mice, suggesting that species-specific differences may play a role in determining the host range of this virus (74). Still, the full-genome transgenic mice are absent of the HBV entry factor NTCP. Although infectious particles are produced, hepatocyte infection does not occur. Furthermore, in human NTCP-expressing transgenic mice, HBV infection in mouse hepatocytes is also limited due to the lack of a host cell dependency factor (100). For these reasons, studies examining HBV entry, mechanisms underlying infection, compounds interfering with the infection process, and cccDNA synthesis are not suitable in full-genome transgenic mice.

## Liver-Humanized Mouse Model

Although mice are not the natural host for HBV, a human liver chimeric mouse model makes it possible to study the entire infection process (from viral entry to cccDNA synthesis and intrahepatic spread). The human liver chimeric mouse model is generated by engraftment of primary human hepatocytes in highly immunodeficient mice. The success of this model is based on the principle of inducing murine hepatocellular damage and abolishing adaptive immune responses to allow survival of the transplanted xenogeneic hepatocytes (10). Three different liver humanized mouse models are currently available for studying HBV infection.

The first kind of liver humanized mouse is the urokinase-type plasminogen activator/severe combined immunodeficiency (alb-uPA/Scid) mouse, in which overexpression of uPA transgene driven by the murine albumin (alb) promoter results in hypofibrinogenemia and severe hepatotoxicity (101). After backcrossing alb-uPA mice with immunodeficient mice (e.g., Scid, RAG2^-/-^ and Scid/beige mice), human hepatocytes are introduced *via* intrasplenic injection and reach the injured liver, where they begin to proliferate to replace the diseased mouse hepatocytes (102). Human hepatocytes can reconstitute up to 70% of liver in this model (103), but it has several drawbacks including kidney and hematologic disorders, potentially fatal bleeding, low breeding efficiency, and narrow time range for liver manipulation (103, 104).

To circumvent these challenges, an alternative human liver chimeric mouse model was generated: the triple knockout FAH^-/-^RAG2^-/-^IL2RG^-/-^ (FRG) mouse. This mouse model was produced by crossing fumaryl acetoacetate hydrolase (Fah) knockout mice with double immunodeficient RAG2^-/-^IL-2 receptor γ chain (IL2RG) mice (105). The Fah gene encodes the last enzyme in the tyrosine catabolism pathway, and its deficiency leads to accumulation of toxic tyrosine metabolic intermediates and subsequent liver failure (106, 107). However, liver injury in this model can be prevented with 2-(2-nitro-4-trifluoromethylethylbenzoyl)-cyclohexane-1,3-dione (NTBC) (105). This model requires continuous cycling of NTBC to sustain human hepatocyte engraftment for extended periods of time, but this can achieve ~97% human liver chimerism (105).

The third mouse model is generated based on TK-NOG mice, which carry the transgene of the herpes simplex virus 1(HSV1) thymidine kinase (TK) in triple immune defect Nod/Scid/IL2rg^-/-^ (NOG) mice (108, 109). Similar to FRG mice, liver injury can be induced in a controlled manner by ganciclovir that selectively destroy TK-expressing murine hepatocytes (108, 109). The liver repopulation rate in this model is ~70%, which is comparable to the alb-uPA/Scid mouse. However, a major drawback is that male TK-NOG mice are sterile.

In contrast to transgenic HBV mice, liver humanized mice are susceptible to HBV infection (110) and cccDNA is formed in transplanted hepatocytes. These models can be used to study viral infection, the nature of cccDNA, and interactions between HBV and its host (111), as well as test novel antiviral agents (112, 113). However, immunodeficiency makes these mice unsuitable for studying immune responses. The high cost and technical difficulties also pose great challenges for the availability of this mouse model.

## Immunocompetent Human Liver Chimeric Mice

Liver humanized mice have complete immunodeficiency, which does not allow the study of adaptive immune responses and immunotherapeutic strategies. To circumvent this limitation, immunocompetent human liver-chimeric mice dually reconstituted with both immune cells and hepatocytes of human origin were developed by transplantation of human hematopoietic stem cells or fetal liver cells (114–117). Another approach to study immune responses in chimeric mice is adoptive transfer of human immune cells into previously infected mice (118). However, the generation of double chimeric systems is still a major challenge; both the source of human cells and mouse background massively influence the outcomes of cell engraftment and maturation.

## The Application of Mouse Models Targeting HBV Cure

Three categories of HBV cure, complete cure, functional cure and partial cure, have been defined according to clinical parameters and the corresponding underlying molecular mechanisms. Complete cure is defined as the loss of HBsAg and the complete elimination of all forms of replicating HBV, including cccDNA while functional cure only refers to HBsAg loss or HBsAg seroconversion (119). Complete cure is an ideal goal; however, current available agents can not completely eradicate the cccDNA in infected hepatocytes. Therefore, a functional cure is the currently accepted goal for new HBV therapies. CHB patients achieving functional cure have a very benign prognosis and the negligible risk of progression to liver cirrhosis and HCC (120).

Current novel treatment options under development targeting HBV functional cure or even HBV eradication included direct antivirals that aim at inhibiting virus entry, degrading cccDNA or preventing cccDNA formation, silencing viral transcripts, modulating nucleocapsid assembly, preventing HBsAg secretion, and immune modulators that aim to restore effective HBV-specific immune responses (121). The liver-humanized mice and immunocompetent human liver chimeric mice allow *de novo* HBV infection and thus maintain the cccDNA pool. Therefore, these two models can be used to test agents targeting HBV entry, cccDNA synthesis, virus assembly and secretion. The rc-cccDNA mouse model is particularly useful for evaluating efficacy of cccDNA-targeting agents. The HDI, Ad-vector and full-genome HBV transgenic mice could be used to evaluate the efficacy of nucleoside analogues and siRNA silencing viral transcripts. Moreover, the immunocompetent mice in HDI system and human liver chimeric mice allows to investigate the restoration of HBV-specific immunity by the treatment with novel immune modulators. As discussed in the previous sections, therapeutic vaccines, toll-like receptor agonists, polymerase inhibitors, small interfering RNAs, and HBV entry inhibitors have been tested as single agents or in combinations in these mouse models. The major advantages of working with mouse models are the ease by which *in vivo* target engagement and the mechanism of actions can be assessed. The wealth of historical data and reagents tested in a great number of diverse mouse studies can be drawn upon to understand the downstream effects on viral infection or the host immunity (122). However, whether success of new treatments in the mouse models could be translated to the human setting remains to be analyzed in each case.

A last critical issue for achieving HBV cure is HBV integration. Unfortunately, this aspect of HBV cure is not yet considered in the investigation in HBV mouse models. Though the transgenic HBV mouse models contain integrated viral genomes in the host genome, the consequence for viral control is not analyzed in an appropriate context and needs attention in the future studies.

## Conclusion

This review discussed the *in vivo* mouse models for HBV research. Each has its own strengths and weaknesses (**Table 3**), offering good opportunities to choose between different models based on the specific research questions addressed. The HDI model can establish both acute and chronic HBV replication in immunocompetent mice, so it is extensively used to investigate immune responses during virus clearance and evaluate novel antiviral agents. When using this model, the mouse background, age, gender, and HBV plasmid dose should be carefully considered. The AAV transduction-mediated replicon delivery model can also establish persistent HBV replication and is used to investigate antiviral compounds. However, due to the non-HBV immunity elicited by the vector, it is still controversial whether this model could be used to study HBV immunology. The development of transgenic mouse models enables elucidation of molecular virus-host interactions and the biology of HBV-related immunology and pathogenesis. The human liver chimeric mouse model also makes it possible to study the entire infection process, but the high cost and technical difficulties limit the wide applicability of this model. In summary, the development of HBV mouse models has opened a new era in HBV research. Together with *in vitro* cell culture models, these mouse models will provide great insight for developing novel therapeutic strategies to cure HBV.

**Table 3:** Characteristics of different types of HBV mouse model.

Mouse models	Advantages	Applications	Limitations
Hydrodynamic injection	Immunocompetent Transient and persistent replication model	Investigate immune responses Testing novel antiviral agents HBV variants or mutants	No cccDNA; No infection; Relatively lower efficiency; Liver damage
**Viral vector-mediated transfection model**			
Ad-HBV transduction	Immunocompetent	Immune-mediated viral clearance	No cccDNA; No infection; High non-HBV immune response; Transient replication
AAV-HBV transduction	Immunocompetent Higher transfection efficiency Persistent replication	Test antiviral agents Study mechanism of fibrosis(?)	cccDNA rarely observed; No infection; immune tolerance
rc-cccDNA mouse model	cccDNA formation	Antiviral agents targeting cccDNA	No infection; not the physical DNA formed by rcDNA
**HBV transgenic mouse model**			
Single protein-transgenic mice	Express single HBV protein	Virology and oncogenic potential of HBV proteins	No infection; No cccDNA No HBV replication
Full genome-transgenic mice	Whole HBV life cycle Effective viral replication	Antiviral drugs interfering HBV replication	No infection; No cccDNA Self-tolerance; Not cytopathic
Liver humanized mouse model	Susceptible to HBV infection cccDNA formation	Study viral infection Interaction between HBV and host	Immune deficient High cost Technical challenging
Immunocompetent human liver chimeric mice	Immunocompetent Susceptible to HBV infection cccDNA formation	Study viral infection Immune responses	High cost Technical challenging

## Author Contributions

YD and ML conceptualized this review. RB, XL, XZ, and JL wrote parts of the review. YD and ML drafted the layout for the review and wrote the discussion. DY edited the review. All authors contributed to the article and approved the submitted version.

## Funding

This work was supported by a scholarship of Medical Faculty of University of Duisburg-Essen, a grant of Deutsche Forschungsgemeinschaft (RTG 1949/2), and grants of National Natural Science Foundation of China (81871664, 81861138044, 91742114 and M-0060).

## Conflict of Interest

The authors declare that the research was conducted in the absence of any commercial or financial relationships that could be construed as a potential conflict of interest.

## Publisher’s Note

All claims expressed in this article are solely those of the authors and do not necessarily represent those of their affiliated organizations, or those of the publisher, the editors and the reviewers. Any product that may be evaluated in this article, or claim that may be made by its manufacturer, is not guaranteed or endorsed by the publisher.
